# Iron deficiency, anemia and association with refugee camp exposure among recently resettled refugees: A Canadian retrospective cohort study

**DOI:** 10.1371/journal.pone.0278838

**Published:** 2022-12-15

**Authors:** Marta B. Davidson, Garielle Brown, Lesley Street, Kerry McBrien, Eric Norrie, Andrea Hull, Rachel Talavlikar, Linda Holdbrook, Gabriel E. Fabreau

**Affiliations:** 1 Division of Hematology and Hematologic Malignancies, Department of Medicine, Cumming School of Medicine, University of Calgary, Calgary, Canada; 2 Department of Community Health Sciences, O’Brien Institute for Public Health, Cumming School of Medicine, University of Calgary, Calgary, Canada; 3 Mosaic Refugee Health Clinic, Mosaic Primary Care Network, Calgary, Canada; 4 Department of Family Medicine, Cumming School of Medicine, University of Calgary, Calgary, Canada; 5 Division of General Internal Medicine, Department of Medicine, Cumming School of Medicine, University of Calgary, Calgary, Canada; Faculty of Medicine, Saint-Joseph University, LEBANON

## Abstract

Malnutrition and poor health are common among recently resettled refugees and may be differentially associated with pre-migration exposure to refugee camp versus non-camp dwelling. We aimed to investigate the associations of iron deficiency (ID), anemia, and ID anemia (IDA) with pre-migration refugee camp exposure among recently arrived refugees to Canada. To this end, we conducted a retrospective cohort study of 1032 adult refugees who received care between January 1, 2011, and December 31, 2015, within a specialized refugee health clinic in Calgary, Canada. We evaluated the prevalence, severity, and predictors of ID, anemia, and IDA, stratified by sex. Using multivariable logistic regression, we estimated the association of refugee camp exposure with these outcomes, adjusting for age, months in Canada prior to investigations, global region of origin, and parity. Among female refugees, the prevalence of ID, anemia, and IDA was 25% (134/534), 21% (110/534), and 14% (76/534), respectively; among males, 0.8% (4/494), 1.8% (9/494), and 0% (0/494), respectively. Anemia was mild, moderate, and severe in 55% (60/110), 44% (48/110) and 1.8% (2/110) of anemic females. Refugee camp exposure was not associated with ID, anemia, or IDA while age by year (ID OR = 0.96, 95% CI 0.93–0.98; anemia OR = 0.98, 95% CI 0.96–1.00; IDA OR = 0.96, 95% CI 0.94–0.99) and months in Canada prior to investigations (ID OR = 0.85, 95% CI 0.72–1.01; anemia OR = 0.81, 95% CI 0.67–0.97; IDA OR = 0.80, 95% CI 0.64–1.00) were inversely correlated with these outcomes. ID, anemia, and IDA are common among recently arrived refugee women irrespective of refugee camp exposure. Our findings suggest these outcomes likely improve after resettlement; however, given proportionally few refugees are resettled globally, likely millions of refugee women and girls are affected.

## Introduction

The world is facing an unprecedented global refugee crisis with an estimated 89.3 million people forcibly displaced from their homes and 27.1 million global refugees [1]. Refugees endure food and housing insecurity, limited healthcare access, and traumatic experiences that negatively impact their health status [2]. Currently, approximately 24% of the world’s refugees reside in camps, and the remaining majority live in urban and rural regions within host-nations [3–5]. Further, high burdens of malnutrition and poor health are common among refugee camp inhabitants [6–8]. The United Nations High Commission for Refugees (UNHCR) now aims to limit camp deployment, instead encouraging integrating refugees within urban host-nation communities whenever possible [3]. It is unclear how these policy changes may affect the nutrition and health of refugees.

Worldwide, iron deficiency (ID) is the most common nutritional deficiency, and anemia is its most commonly associated adverse health outcome [9–14]. ID and iron deficiency anemia (IDA) are most commonly caused by inadequate access to iron-rich foods or poor iron absorption [15]. In women and adolescent girls, ID is also linked to menstrual blood loss and high parity [16]. Further, enteric pathogens from exposures to contaminated drinking water or foods can cause chronic intestinal blood loss leading to ID [17]. Globally, anemia has been linked to poor pregnancy outcomes [18, 19], reduced work productivity [20, 21], increased morbidity and increased all-cause mortality [22]. Refugees residing in refugee camps experience high prevalence of anemia and nutritional deficiencies [23], especially among women and children [10, 24–28]. It is currently unclear, however, if refugees in non-camp settings experience similar rates of nutritional deficiencies and anemia.

We aimed to characterize the prevalence and severity of ID, anemia, and IDA among recently-arrived adult refugees and claimants with and without pre-migration refugee camp exposure, and its differential association with these outcomes. We hypothesized that recently resettled refugees and asylum claimants exposed to refugee camps have higher prevalence and severity of ID, anemia, and IDA due to increased nutritional deficiencies compared to refugees living outside of refugee camps. Finally, we also aimed to characterize anemia unrelated to ID in this population.

## Methods

### Setting and data sources

We performed a retrospective cohort study among recently arrived refugees who presented to the *Mosaic Refugee Health Clinic* (MRHC) in Calgary, Canada. The MRHC is the only specialized refugee health clinic in its geographic area and provides multidisciplinary primary and specialty care to refugees and asylum claimants (claimants) for up to two years after resettlement. We performed a comprehensive chart review of electronic medical records (EMRs) using combined manual and automated methods to collect detailed sociodemographic, resettlement, clinical, biometric, laboratory, and clinic utilization data.

### Study population

We included all refugees and claimants, herein referred to collectively as “refugees”, presenting to MRHC between January 1, 2011 and December 31, 2015 who: (1) were at least 18 years old at clinic intake, (2) had at least 2 clinic appointments, and (3) completed relevant laboratory investigations within 180 days of arrival to Canada, to investigate the associations of pre-migration refugee camp exposure with nutritional status and our outcomes of interest. We excluded subjects missing important data.

### Study variables

In Canada, convention refugees are defined as “a person who meets the refugee definition in the 1951 Geneva Convention relating to the Status of refugees [29], and are classified as either government assisted refugees (GAR) or privately sponsored refugees (PSR) [30]. We defined claimants as either subjects that were seeking protection in Canada but had not yet received convention refugee status, or those who had their refugee claims rejected during the study period. We extracted sociodemographic data including sex, date of birth, country of origin, refugee category, and date of arrival to Canada from EMR-recorded immigration documents. We categorized country of origin according to the United Nations’ Geoscheme [31], and defined it as either a subject’s country of birth, citizenship or inhabitation prior to migration, depending on where they lived the longest. We considered refugee camp exposure present if it was recorded in the intake history, regardless of duration. Two chart abstractors followed a standardized protocol to manually abstracted each study subject’s clinical information from the EMR and categorize clinical diagnoses into ICD-10 codes [32]. Agreement between chart abstractors was >90% for clinical diagnostic codes; disagreements were resolved by a senior clinician. Among female subjects, pregnancy status and number of live children were recorded at intake. We considered subjects pregnant if pregnancy was confirmed at the time of laboratory investigations and assumed parity to be zero if it was not explicitly recorded in the EMR.

### Study outcomes

We analyzed laboratory data to identify our primary outcomes of interest: ID, anemia, and IDA. Anemia was defined according to World Health Organization (WHO) definitions as: a hemoglobin <120g/L for all non-pregnant females ≥15 years of age, <110g/L for pregnant females ≥15 years of age, and <130g/L for males ≥15 years of age [33]. Among anemic refugees, we assessed anemia severity and morphology. We defined mild, moderate, and severe anemia among non-pregnant women as a hemoglobin concentration of: 110-119g/L, 80-109g/L, and <80g/L, respectively, and among pregnant women as 100-109g/L, 70-99g/L, and <70g/L, respectively [33]. In adult males ≥18 years of age, we defined mild, moderate, and severe anemia as a hemoglobin concentration of 110-129g/L, 80-109g/L, and <80g/L, respectively [33]. For all subjects we further classified anemia as: microcytic (<77 femtolitres [fl]), normocytic (77–96fl), or macrocytic (>96fl) [34]. Finally, we defined iron deficiency (ID) according to WHO standards as a serum ferritin concentration of <15μg/L [35, 36] and IDA as the concomitant presence of anemia and ID as outlined above.

### Missing data

Fifty-six individuals were missing data for “number of children”, which was replaced with the most frequent and median number of children: two. We tested this assumption by replacing these 56 missing data points with zero, which did not change any study outcomes.

### Statistical analysis

We stratified our analysis by sex given the known variation in prevalence of iron deficiency and anemia with sex [24]. We reported descriptive statistics according to refugee camp exposure, stratified by sex. We reported normally-distributed continuous variables as means with standard deviations (SD), and all non-normally distributed variables as medians with interquartile ranges [IQR]. We assessed statistical differences among demographic factors by refugee camp exposure using the Wilcoxon rank sum tests for non-normally-distributed variables, and the chi-square or Fisher’s exact tests for normally distributed variables.

We used univariate logistic regression models to estimate unadjusted odds ratios for the outcomes of interest, comparing refugees with and without camp exposure. Given the very low prevalence of anemia among males, we restricted our adjusted analyses to women only.

We initially utilized multivariable, two-level, logistic regression models with a random effect for UN region of origin, to account for non-random clustering of patients according to global regions and their associations with our outcomes of interest [19]. We allowed the UN region intercepts to vary randomly among patients (level 1) within similar UN global regions of origin (level 2). We then investigated the degree of patient clustering within global regions and sub-regions and its association with the variances observed for our outcomes of interest. The intraclass correlation coefficients was 0.001 for all three outcomes, indicating a negligible degree of clustering within global regions and sub-regions thus negating the need for multi-level regression models. We thus used multivariable logistic regression models to estimate the adjusted odds for the outcomes of interest among female refugees adjusting for refugee camp exposure, age, UN global region of origin (Africa as reference group), number of months in Canada prior to laboratory investigations, active pregnancy status (for ID), and number of children (parity).

### Sensitivity analysis

To test our definition of ID based on serum ferritin concentration, we performed a sensitivity analysis whereby we assessed our outcomes of interest using ID defined as a transferrin saturation (tsat) of less than 0.16 [33] and IDA as anemia with a concomitant tsat <0.16.

### Exploratory analysis

For all anemia cases not associated with ID (ferritin <15g/L), we explored patient records for alternate etiologies of anemia and characterized these conditions by ICD-10 diagnostic codes. We then classified these conditions into the following categories: B12 deficiency, thalassemic conditions, ID, and other if patients did not have any anemia-specific ICD-10 codes documented within 6 months of arrival to Canada. ID or IDA diagnoses were excluded if the patient’s ferritin concentration was above 100g/L, a level largely considered adequate to rule out ID even in context of inflammatory states [37]. In these cases, we sought other anemia-diagnoses from patients’ charts.

All analyses were performed using Stata 14 (College Station, TX USA: StataCorp LLC) and SAS 9.4 (Cary, NC, USA: SAS Institute Inc.) and considered a 2-tailed alpha <0.05 as statistically significant. This study was approved by the University of Calgary Research Ethics Board.

## Results

### Baseline characteristics

**Fig 1:**
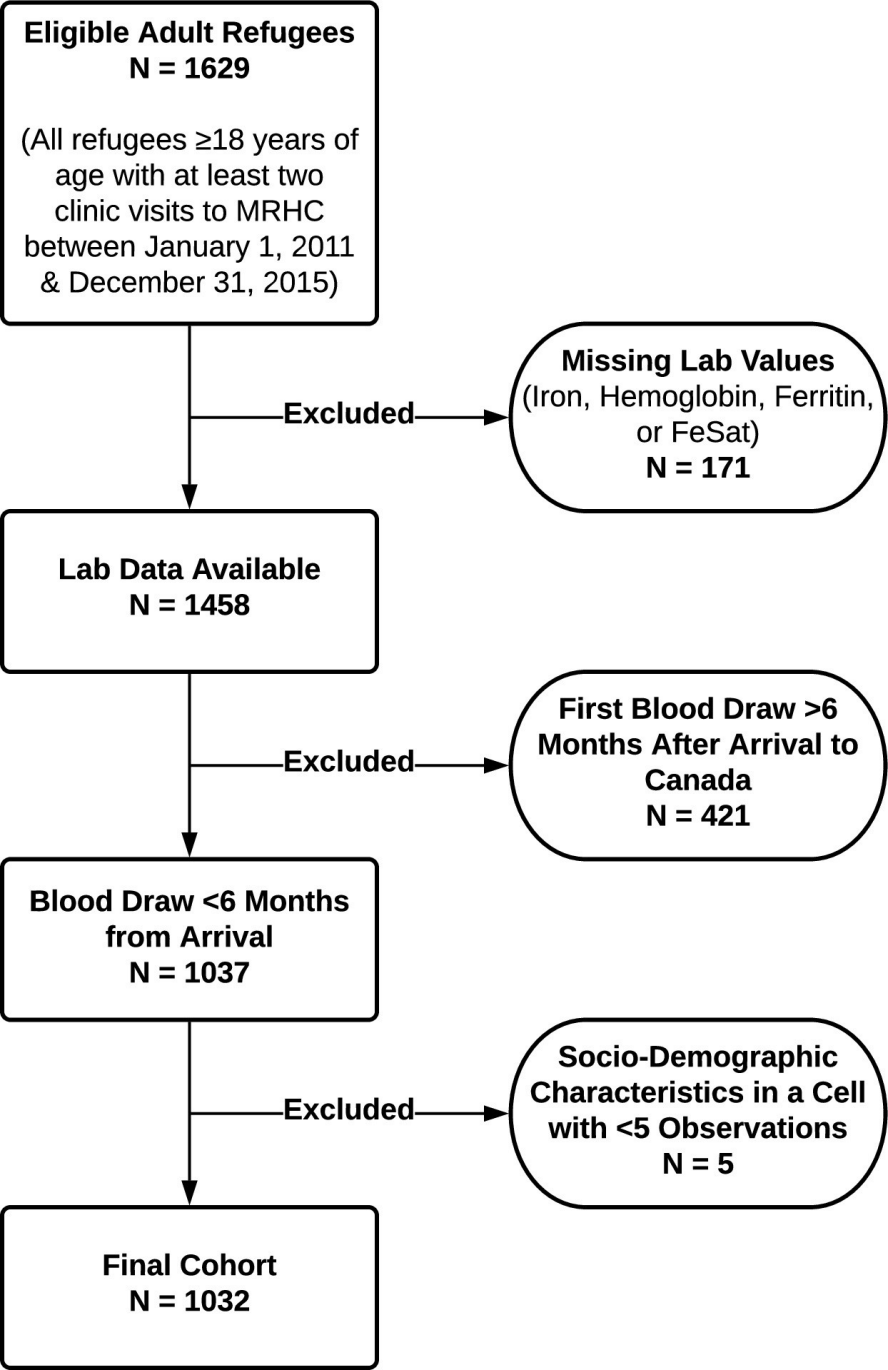
Flow chart of study. See text for details.

We identified 1032 adult refugee patients in our cohort, with 52% (534/1032) females and 48% (498/1032) males (Fig 1). The median age was 32.6 years [26.3–41.3] and 31.2 years [25.2–39.8] among males and females, respectively. Subjects originated from 47 different countries. The top five countries of origin were Eritrea (23%), Iraq (14%), Ethiopia (13%), Afghanistan (6%) and Bhutan (6%). Among UN global regions, 53% (542/1032) of refugee patients were from Africa, 45% (461/1032) were from Asia, 2% (22/1032) were from the Americas, and 1% (10/1032) were from Europe (Table 1). Overall, 18.7% (193/1032) of refugees were exposed to a refugee camp, with African and government assisted refugees (GAR) overrepresented among those exposed. Among females, English proficiency varied by refugee camp exposure, but pregnancy rates and number of children did not (Table 1).

**Table 1:** Patient characteristics by sex and refugee camp exposure (N = 1032).

Variable	Female N = 534			Male N = 498		
	Refugee Camp +	Refugee Camp -	p-value*	Refugee Camp +	Refugee Camp -	p-value*
	N = 94	N = 440		N = 99	N = 399	
**Age** (years)–Median [IQR]	30.3 [23.6–38.3]	31.4 [25.5–40.0]	0.28	32.0 [25.5–42.4]	32.7 [26.5–41.1]	0.92
**Months in Canada Prior to Blood Tests–**Median [IQR]	1.1 [0.7–1.9]	1.4 [0.8–2.5]	0.08	1.2 [0.8–2.1]	1.3 [0.7–2.6]	0.69
**UN Global Region–**N (%)						
Africa	63 (67.0)	206 (46.8)	< 0.01	69 (69.7)	204 (51.1)	< 0.01
Americas	0 (0)	17 (3.9)		0 (0)	5 (1.3)	
Asia	31 (33)	210 (47.7)		30 (30.3)	190 (47.6)	
Europe	0 (0)	7 (1.6)		0 (0)	0 (0)	
**Number of Children–**Median [IQR]	1 [0–4]	2 [0–3]	0.43	1 [0–2]	1 [0–2]	0.86
**Pregnancy**^**^**^–N (%)						
Yes	6 (6.4)	48 (10.9)	0.19		N/A	
No	88 (93.6)	392 (89.1)				
**Refugee Category** ^#^						
GAR	71 (75.5)	204 (46.4)	< 0.01	65 (65.7)	188 (47.1)	< 0.01
PSR	23 (24.5)	191 (43.4)		34 (34.3)	180 (45.1)	
Claimant	0 (0)	45 (10.2)		0 (0)	31 (7.8)	
**English Language proficiency at intake–**N (%)						
None	61 (64.9)	214 (48.6)		38 (38.4)	129 (32.3)	
Limited	11 (11.7)	73 (16.6)		20 (20.2)	86 (21.6)	
Good	22 (23.4)	153 (34.8)	0.02	41 (41.4)	184 (46.1)	0.52

*p-values calculated using a Wilcoxon rank sum test for comparison of medians and chi-square or Fisher’s exact tests for comparison of frequencies.

#GAR refer to Government assisted refugees, PSR refers to privately sponsored refugees, and Claimant refers to refugee claimants or asylum seeker.

^Pregnant refers to active pregnancy at the time of laboratory investigations. N/A refers to not applicable.

### Prevalence of ID, anemia, and IDA among refugees

**Fig 2:**
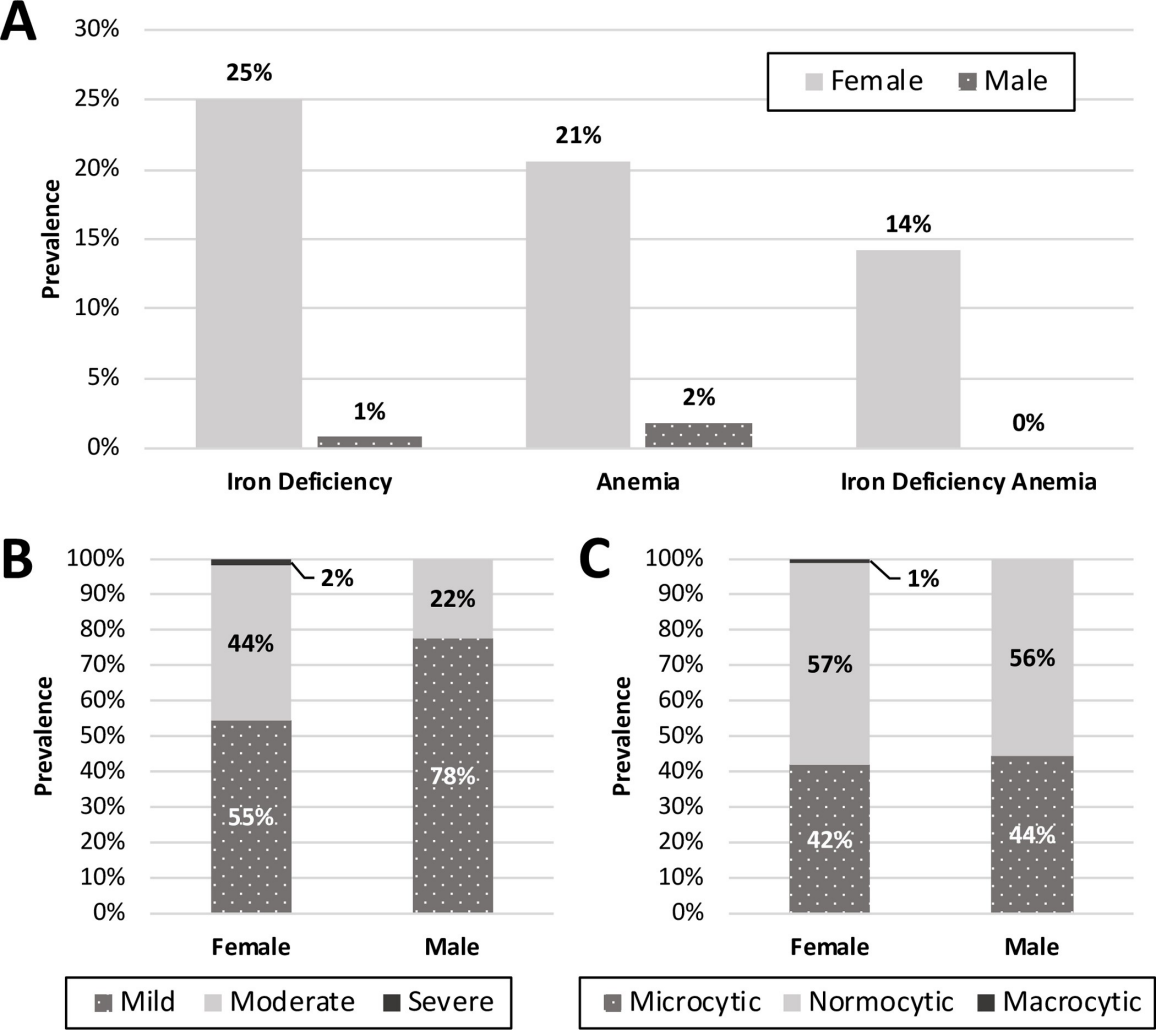
Iron deficiency and anemia characteristics in adult refugees resettled to Calgary, Alberta. (A) Prevalence of ID, anemia, and IDA in male and female refugees. We defined ID as a serum ferritin <15μg/L and anemia according to the WHO standards [15]. (B) Anemia severity in male and female refugees. Among non-pregnant women we defined mild, moderate, and severe anemia as a hemoglobin concentration of 110-119g/L, 80-109g/L, and below 80g/L, respectively, and 100-109g/L, 70-99g/L, and <70g/L, respectively among pregnant women. In adult males ≥18 years of age, we defined mild, moderate, and severe anemia as hemoglobin concentrations of 110-129g/L, 80-109g/L, and <80g/L respectively. (C) Anemia morphology. Anemia was classified as microcytic (<77fl), normocytic (77–96fl), or macrocytic (>96fl).

Among all subjects, the median time from arrival to Canada to first laboratory investigations was 39 days [22–72 days]. Among females and males, the prevalence of ID was 25% (134/534) and 0.8% (4/498), respectively (p<0.01) (Fig 2A), the median ferritin level was 29μg/L [14–57μg/L] and 120.5μg/L [70–186μg/L] (p<0.01), respectively, and the prevalence of anemia was 21% (110/534) and 1.8% (9/498), respectively (p<0.01). Sixty-nine percent (76/110) of anemia among females was attributed to ID. There were no IDA cases among males. Among anemic females, 55% (60/110) had mild, 44% (48/110) had moderate, and 2% (2/110) had severe anemia. Among anemic males, 78% (7/9) had mild, 22% (2/9) had moderate, and none had severe anemia (Fig 2B). Among anemic subjects, most anemia was normocytic and only one female had macrocytic anemia (Fig 2C).

#### Unadjusted outcomes

Table 2 summarizes the prevalence of ID, anemia, and IDA among all subjects by refugee camp exposure stratified by sex. There were no prevalence differences in the outcomes of interest by refugee camp exposure. In our unadjusted analysis restricted to females (Table 3), refugee camp exposure, UN region of origin, and number of children were not associated with any of the outcomes of interest; however, each increased year of age and each month in Canada prior to laboratory investigations were inversely associated with these outcomes. Pregnancy was associated with ID but was not tested against anemia or IDA as we used pregnancy specific definitions for these outcomes (Table 3).

**Table 2:** Primary outcomes by sex and refugee camp exposure (N = 1032).

Variable	Female N = 534			Male N = 498		
	Refugee Camp +	Refugee Camp -	p-value*	Refugee Camp +	Refugee Camp -	p-value*
	N = 94	N = 440		N = 99	N = 399	
**Iron Deficiency–N (%)**	19 (20.2)	115 (26.1)	0.24	0 (0)	4 (1.0)	0.99
**Anemia–N (%)**	19 (20.2)	91 (20.7)	0.99	4 (4.0)	5 (1.3)	0.08
**Iron Deficiency Anemia–N (%)**	12 (12.8)	64 (14.6)	0.75	0 (0)	0 (0)	–

**Table 3:** Unadjusted outcomes for females (N = 534).

Variables	Iron Deficiency	Anemia	Iron Deficiency Anemia
	Unadjusted Odds Ratio [95% CI]	Unadjusted Odds Ratio [95% CI]	Unadjusted Odds Ratio [95% CI]
**Refugee Camp Exposure**	0.72 [0.41–1.24]	0.97 [0.56–1.69]	0.86 [0.44–1.67]
**Age (in years) at First Appointment**	**0.97 [0.95–0.99]**	0.98 [0.96–1.00]*	**0.96 [0.94–0.99]**
**Months in Canada Prior to Blood Tests**	**0.83 [0.71–0.97]**	**0.83 [0.70–0.99]**	0.82 [0.66–1.00]**
**UN Global Region**	Calculated vs. Africa	Calculated vs. Africa	Calculated vs. Africa
Americas	0.75 [0.21–2.68]	0.47 [0.11–2.13]	0.38 [0.05–2.95]
Asia	1.37 [0.92–2.05]	0.86 [0.56–1.33]	1.03 [0.63–1.70]
Europe	2.61 [0.57–12.00]	1.42 [0.27–7.53]	2.43 [0.46–12.99]
**Number of Children**	0.99 [0.90–1.10]	0.93 [0.83–1.04]	0.91 [0.79–1.04]
**Pregnant** ^^^	**1.89 [1.05–3.41]**	N/A	N/A

*Results statistically significant, p = 0.012.

**Results statistically significant, p = 0.04.

^Pregnant refers to active pregnancy at the time laboratory investigations were drawn.

### Adjusted analysis

**Fig 3:**
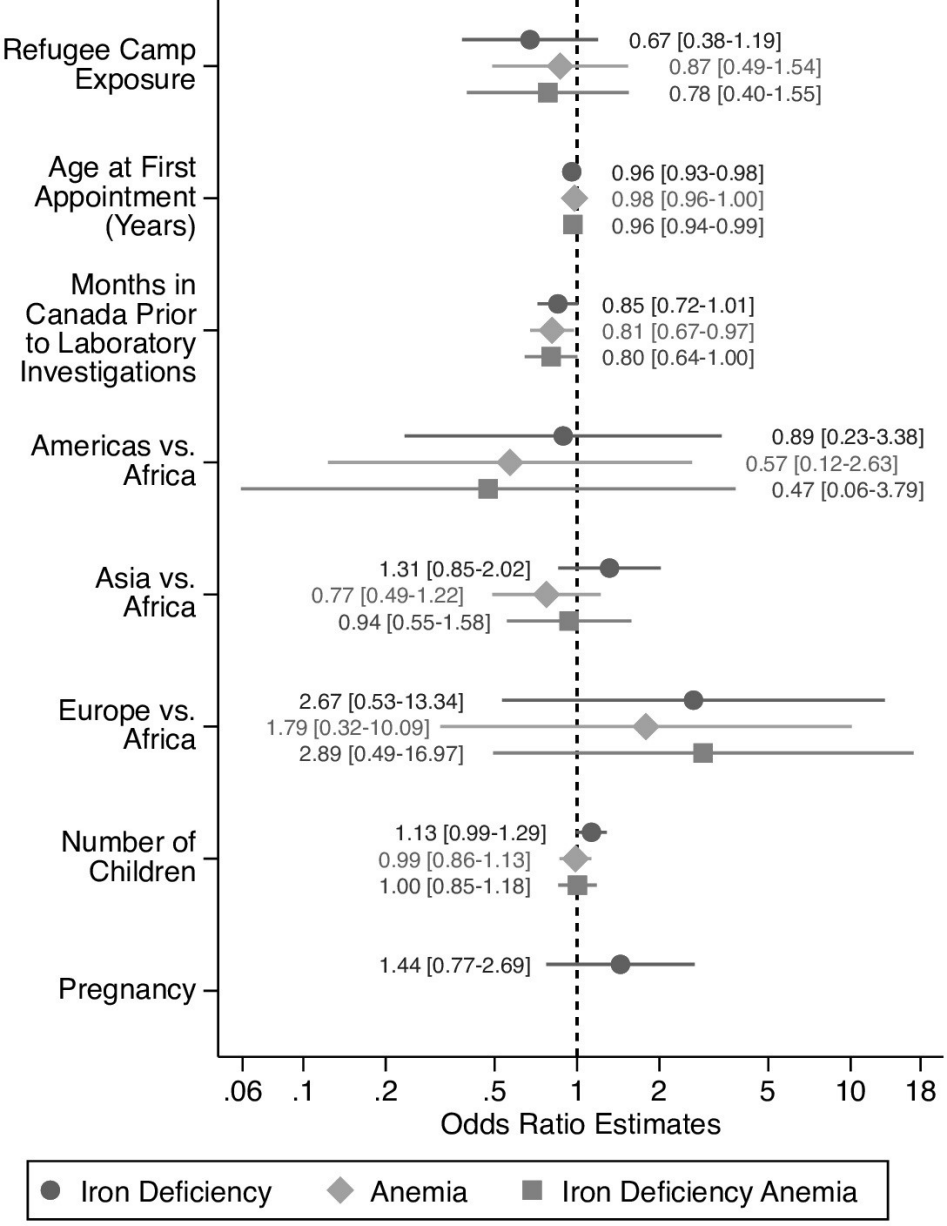
Predictors of ID, Anemia, and IDA in recently resettled global female refugees. Multi-variable logistic regression was used to estimate the odds of ID, anemia, and IDA in female refugees after adjusting for refugee camp exposure, age, global region of origin (defined by UN global regions), pregnancy status and number of children. (*) indicates p-value <0.05.

In our adjusted analysis, refugee camp exposure was not associated with our outcomes of interest among female refugees (Fig 3). Further, UN region of origin, pregnancy, and number of children were also not associated with ID, anemia, or IDA. Each increased year of age at first clinic appointment was associated with 4% and 3% decreased odds of ID and IDA, respectively (Fig 3). Finally, each month in Canada prior to when laboratory investigations were drawn was associated with 19% and 20% decreased odds of anemia and IDA, respectively (Fig 3).

### Sensitivity analysis

In our sensitivity analysis, the overall prevalence of ID obtained using transferrin saturation (tsat) [21.5% (222/1032)] was higher than using a ferritin-based definition [13.3% (138/1032)] (S1 Table). However, this did not change the results of our unadjusted (S2 Table) or adjusted analysis (S1 Fig).

### Exploratory analysis: Determinants of non-iron deficiency associated anemia

**Fig 4:**
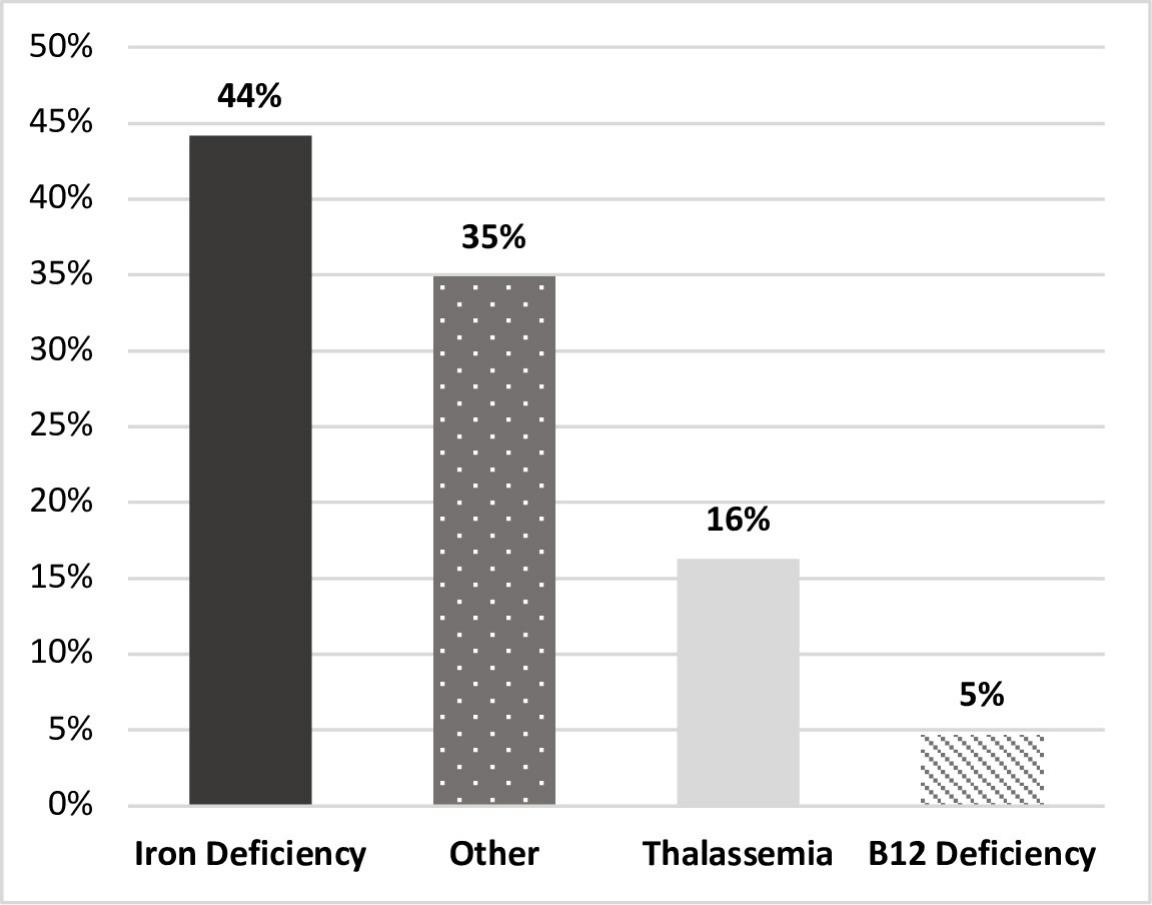
Etiology of anemia not attributable to iron deficiency. Conditions causing anemia using ICD10 diagnostic codes extracted from charts among patients with anemia, but a serum ferritin concentration >15μg/L (N = 43).

Among anemia cases, 36% (43/119) were not attributable to ID as defined in our study. In these 43 non-ID subjects, physicians assigned diagnoses of IDA in 44% (19/43), thalassemia trait in 16% (7/43), and B12 deficiency in 5% (2/43) of individuals (Fig 4). Thirty-five percent (15/43) of these individuals did not have an explicit cause of anemia identified (Fig 4). Among patients with physician-assigned diagnoses of IDA, the median ferritin was 22.5μg/L [17–33μg/L]. Two patients were misclassified as having IDA by physician-assigned diagnoses as each had ferritin concentrations of >100μg/L; we therefore excluded these cases from median ferritin calculations. When we combined physician assigned cases of IDA with laboratory-defined cases, ID accounted for 80% (95/119) of anemia cases in our cohort.

## Discussion

Among a global cohort of adult refugees recently arrived in Canada, iron deficiency (ID), anemia, and iron deficiency anemia (IDA) were highly prevalent conditions irrespective of pre-migration refugee camp exposure. This was especially notable among female refugees, in whom the prevalence of ID was almost three times higher than that reported for adult Canadian females [38]. Contrary to our hypothesis, ID, anemia, and IDA were not associated with previous refugee camp exposure. However, we found that 82% of anemia cases among adult refugees were attributable to nutritional deficiencies (Iron or B12), and that inherited red blood cell disorders or other causes of anemia were rare. Given that refugees in our cohort completed laboratory investigations with a median time of 39 days after arrival to Canada, our findings likely reflect pre-migration hematologic indices, as well as nutritional and health status.

The 21% anemia prevalence among refugee women in our study is consistent with previous reports [10, 25, 26, 39], and indicates a condition of moderate public health significance by WHO criteria [15]. We found female refugees had a 31-fold higher prevalence of ID and an 11-fold higher prevalence of anemia compared to males [24], consistent with higher anemia frequencies among women reported globally [10, 25, 26, 39]. We found that 25% of refugee women were iron deficient, which is likely an underestimate as we used a stringent ferritin concentration threshold of 15μg/L to define ID that likely misses milder, but clinically relevant deficiencies [40, 41]. Using our stringent ID definition, we found that almost 70% of anemic females had laboratory confirmed ID. Moreover, when we included physician assigned IDA cases with a median ferritin concentration of 22.5μg/L, ID accounted for 84% of observed anemia among women. This further suggests that our estimates are conservative, and that refugee women are at high risk for ID and anemia and their associated adverse health outcomes, irrespective of refugee camp exposure or global region or origin.

To our knowledge, this is the first study to investigate the association of nutritional and health status with pre-migration refugee camp exposure among global refugees, from 47 different countries, resettled to a high-income country. Overall, we found no differences in the prevalence of ID, anemia, and IDA among refugees by refugee camp exposure. Our findings suggest that refugees in non-camp settings (often in urban settings) may experience similar nutritional deficiencies as well as barriers accessing food and nutritional supports as camp-dwelling refugees. A recent systematic review also showed that nutritional deficiencies are a significant problem among non-camp dwelling refugees [42]. A number of factors may be contributing to this problem. For example, non-camp refugees may lack access to supports from the World Food Programme [43] and other international aid organizations that traditionally support refugees in camp settings. Further, the majority of displaced persons are relocated to lower income countries [1, 42], which likely have limited resources to offer to urban-dwelling refugees. Our findings appear to contradict the purported health and economic benefits of non-camp dwelling for refugees cited by the UNHCR such as greater freedom of movement and access to employment, given that nutritional deficiencies and food access barriers are most often related to poverty [3].

While high rates of ID and anemia have been documented in selected refugee camps [23], refugee camp exposure has been variably associated with anemia in the literature. For example, among pregnant Sudanese refugees in Ethiopia, anemia was inversely associated with duration of residence in a refugee camp [44]. By contrast, Syrian refugee women in refugee camps in Jordan, but not in Lebanon, were more likely to be anemic than their non-camp counterparts [6, 45]. In Southeast Asia, among refugees from Vietnam, Laos, and Cambodia, there was no association between anemia and refugee camp residence after accounting for ethnicity [19]. These observed differences may be due to the geographic and cultural variability between different refugee camps, as well as significant differences in their availability of nutrition supports and medical services. Unlike previous studies that have been limited to specific global sub-regions and individual ethnocultural populations, our study investigated a global cohort of refugees across 47 countries of origin. We found negligible clustering by UN global region of origin for ID, anemia, and IDA, suggesting that the associations of interest were not significantly different across global regions. Together our findings suggest that ID, often related to food insecurity, is highly prevalent in pre-menopausal refugee women irrespective of pre-migration living situation or global origin.

Our study has limitations that warrant consideration. As our study was conducted at a single site, it may not be representative of refugee populations in other host-regions. Further, our study enrolment ended prior to the influx of approximately 41,000 Syrian refugees to Canada in 2015–2016, and more recent Afghan and Ukrainian refugees [46, 47], thus, it may not represent the current global refugee population. While our study is amongst the largest Canadian refugee cohort studies conducted to-date and comprised a heterogeneous global refugee cohort from 47 different countries, it is underpowered to assess country-specific variations which may factor into the lack of significant regional or sub-regional variation in our outcomes. Moreover, we only identified two cases of severe anemia in our study and may underestimate the prevalence of severe anemia cases that may present to acute care settings. This may be due to a selection bias given that our study relied on outpatient laboratory tests. Alternatively, selection pressures during the refugee migration process may select against individuals with severe anemia who may succumb to its complications or be unable to travel [48]. In addition, because our study was restricted to adult refugees and claimants, post-menarche adolescent girls, who likely also experience high prevalence of ID, anemia, and IDA were not included, thus potentially underestimating their prevalence. Finally, in our cohort, 19% of refugees had resided in a refugee camp prior to arrival in Canada, lower than current global trends, where 24% of global refugees reside in camps [4], suggesting camp-exposure may be underrepresented in our cohort.

Taken together, our findings, in concordance with other global data, suggest that refugee women commonly experience ID and IDA, and that refugee status itself, irrespective of pre-migration dwelling, region of origin, or political refugee class predicts these nutritional deficiency-related conditions. Our findings support the current *Canadian Collaboration for Immigrant and Refugee Health* (CCIRH) screening guidelines, which recommend screening all female refugees, but not males for anemia and ID [49]. In our cohort, anemia varied inversely with months spent in Canada prior to laboratory investigations, suggesting that it may be largely remediable in food-secure environments and with access to high quality primary healthcare.

The unprecedented number of forcibly displaced people globally highlights the scale of the population at risk for nutritional deficiencies. Further, the magnitude of the burden of ID and IDA among displaced women and adolescent females likely reaches at least a moderate public health concern by WHO criteria and warrants consideration of large-scale action to prevent adverse outcomes of this readily preventable nutritional deficiency. Globally, less than 2% of refugees are resettled in high income countries such as Canada; however, studies among multinational cohorts of recently resettled refugees can improve understanding of health conditions faced by refugees regionally and universally [50]. Future studies of ID and anemia among global refugees would help to elucidate if regional and cultural differences exist, and if their prevalence varies across different nutritional and medical assistance models between refugee camps. Improved understanding of nutritional deficiencies in refugee populations may inform treatment and prevention programs, such as targeted iron supplementation programs for pre-menopausal refugee women and adolescent girls.

## Supporting information

S1 Fig Sensitivity analysis. For sensitivity analysis, ID was defined as a serum transferrin saturation (tsat) ≤ 0.16 and IDA was defined as the presence of anemia as defined by WHO thresholds in combination with a tsat ≤ 0.16. Multi-variable logistic regression was used to estimate the odds of ID, anemia, and IDA in female refugees after adjusting for refugee camp exposure, age, global region of origin (defined by UN global regions), pregnancy status and number of children.
(TIF)

S1 Table Sensitivity analysis; primary outcomes by sex and refugee camp exposure using serum transferrin saturation (tsat) (N = 1032). (DOCX)

S2 Table Sensitivity analysis: Unadjusted outcomes among female refugees using transferring saturation (tsat) (N = 534). (DOCX)

## References

[1] UNHCR. Global Trends Report: Forced Displacement in 2021: The UN Refugee Agency; 2022 [cited 2022. Available from: https://www.unhcr.org/62a9d1494/global-trends-report-2021].

[2] Schilling T, Rauscher S, Menzel C, Reichenauer S, Muller-Schilling M, Schmid S, et al. Migrants and Refugees in Europe: Challenges, Experiences and Contributions. Visc Med. 2017;33(4):295–300. doi: 10.1159/000478763 29034258 PMC5624244

[3] UNHCR. Alternatives To Camps 2014 [Available from: http://www.unhcr.org/protection/statelessness/5422b8f09/unhcr-policy-alternatives-camps.html].

[4] UNHCR. Diagnostic Tool for Alternatives to Camps 2017 Global Results 2017.

[5] UNHCR. Shelter: The UN Refugee Agency; 2022 [Available from: https://www.unhcr.org/shelter.html].

[6] Bilukha OO, Jayasekaran D, Burton A, Faender G, King’ori J, Amiri M, et al. Nutritional status of women and child refugees from Syria-Jordan, April-May 2014. MMWR Morb Mortal Wkly Rep. 2014;63(29):638–9. 25055188 PMC5779421

[7] Lutfy C, Cookson ST, Talley L, Rochat R. Malnourished children in refugee camps and lack of connection with services after US resettlement. J Immigr Minor Health. 2014;16(5):1016–22. doi: 10.1007/s10903-013-9796-6 23430464 PMC4830467

[8] Seal AJ, Creeke PI, Mirghani Z, Abdalla F, McBurney RP, Pratt LS, et al. Iron and vitamin A deficiency in long-term African refugees. J Nutr. 2005;135(4):808–13. doi: 10.1093/jn/135.4.808 15795439

[9] Redditt VJ, Janakiram P, Graziano D, Rashid M. Health status of newly arrived refugees in Toronto, Ont: Part 1: infectious diseases. Can Fam Physician. 2015;61(7):e303–9. 26175381 PMC4501620

[10] Redditt VJ, Graziano D, Janakiram P, Rashid M. Health status of newly arrived refugees in Toronto, Ont: Part 2: chronic diseases. Can Fam Physician. 2015;61(7):e310–5. 26175382 PMC4501621

[11] Tiong AC, Patel MS, Gardiner J, Ryan R, Linton KS, Walker KA, et al. Health issues in newly arrived African refugees attending general practice clinics in Melbourne. Med J Aust. 2006;185(11–12):602–6. doi: 10.5694/j.1326-5377.2006.tb00724.x 17181501

[12] Stellinga-Boelen AA, Storm H, Wiegersma PA, Bijleveld CM, Verkade HJ. Iron deficiency among children of asylum seekers in the Netherlands. J Pediatr Gastroenterol Nutr. 2007;45(5):591–5. doi: 10.1097/MPG.0b013e31810e76a5 18030238

[13] Salehi L, Lofters AK, Hoffmann SM, Polsky JY, Rouleau KD. Health and growth status of immigrant and refugee children in Toronto, Ontario: A retrospective chart review. Paediatr Child Health. 2015;20(8):e38–42. doi: 10.1093/pch/20.8.e38 26744562 PMC4699522

[14] Paxton GA, Sangster KJ, Maxwell EL, McBride CR, Drewe RH. Post-arrival health screening in Karen refugees in Australia. PLoS One. 2012;7(5):e38194. doi: 10.1371/journal.pone.0038194 22693599 PMC3364970

[15] De Benoist B, McLean E, Egli I, Cogswell M. Worldwide prevalence of anaemia 1993–2005: WHO global database on anaemia. 2008. Geneva: World Health Organization Google Scholar. 2011.

[16] Breymann C, Auerbach M. Iron deficiency in gynecology and obstetrics: clinical implications and management. Hematology Am Soc Hematol Educ Program. 2017;2017(1):152–9. doi: 10.1182/asheducation-2017.1.152 29222250 PMC6142528

[17] Tolentino K, Friedman JF. An update on anemia in less developed countries. Am J Trop Med Hyg. 2007;77(1):44–51. 17620629

[18] Scholl TO, Hediger ML. Anemia and iron-deficiency anemia: compilation of data on pregnancy outcome. Am J Clin Nutr. 1994;59(2 Suppl):492S-500S discussion S-1S. doi: 10.1093/ajcn/59.2.492S 8304287

[19] Catanzaro A, Moser RJ. Health status of refugees from Vietnam, Laos, and Cambodia. JAMA. 1982;247(9):1303–8. 7062547

[20] Ohira Y, Edgerton VR, Gardner GW, Gunawardena KA, Senewiratne B, Ikawa S. Work capacity after iron treatment as a function of hemoglobin and iron deficiency. J Nutr Sci Vitaminol (Tokyo). 1981;27(2):87–96. doi: 10.3177/jnsv.27.87 7310553

[21] Edgerton VR, Ohira Y, Hettiarachchi J, Senewiratne B, Gardner GW, Barnard RJ. Elevation of hemoglobin and work tolerance in iron-deficient subjects. J Nutr Sci Vitaminol (Tokyo). 1981;27(2):77–86. doi: 10.3177/jnsv.27.77 7310552

[22] Martinsson A, Andersson C, Andell P, Koul S, Engstrom G, Smith JG. Anemia in the general population: prevalence, clinical correlates and prognostic impact. Eur J Epidemiol. 2014;29(7):489–98. doi: 10.1007/s10654-014-9929-9 24952166

[23] Refugees UNHCF. UNHCR Strategic Plan for Anaemia Prevention, Control and Reduction. Reducing the global burden of anaemia in refugee populations 2008–2010. 2010.

[24] Kassebaum NJ, Collaborators GBDA. The Global Burden of Anemia. Hematol Oncol Clin North Am. 2016;30(2):247–308. doi: 10.1016/j.hoc.2015.11.002 27040955

[25] Johnston V, Smith L, Roydhouse H. The health of newly arrived refugees to the Top End of Australia: results of a clinical audit at the Darwin Refugee Health Service. Aust J Prim Health. 2012;18(3):242–7. doi: 10.1071/PY11065 23069368

[26] Ouimet MJ, Munoz M, Narasiah L, Rambure V, Correa JA. [Current pathologies among asylum seekers in Montreal: prevalence and associated risk factors]. Can J Public Health. 2008;99(6):499–504. 19149395 10.1007/BF03403785 PMC6975710

[27] Ahmed RH, Yussuf AA, Ali AA, Iyow SN, Abdulahi M, Mohamed LM, et al. Anemia among pregnant women in internally displaced camps in Mogadishu, Somalia: a cross-sectional study on prevalence, severity and associated risk factors. BMC Pregnancy Childbirth. 2021;21(1):832. 34906104 10.1186/s12884-021-04269-4 PMC8670163

[28] Engidaw MT, Wassie MM, Teferra AS. Anemia and associated factors among adolescent girls living in Aw-Barre refugee camp, Somali regional state, Southeast Ethiopia. PLoS One. 2018;13(10):e0205381. doi: 10.1371/journal.pone.0205381 30308060 PMC6181359

[29] Refugees CCf. Refugees and Immigrants: A glossary [Available from: http://ccrweb.ca/en/glossary].

[30] Canada Go. Terms and definitions related to refugee protection. 2019 [Available from: https://www.canada.ca/en/immigration-refugees-citizenship/corporate/publications-manuals/operational-bulletins-manuals/refugee-protection/terms-definitions-related-refugee-protection.html].

[31] United Nations SD. 2018 [Available from: https://unstats.un.org/unsd/methodology/m49/].

[32] International Statistical Classification of Diseases and Related Health Problems 10th Revision [Available from: http://apps.who.int/classifications/icd10/browse/2010/en].

[33] Haemoglobin concentrations for the diagnosis of anaemia and assessment of severity. [Internet]. World Health Organization. 2011. Available from: https://www.who.int/vmnis/indicators/haemoglobin.pdf.

[34] Brugnara C, Mohandas N. Red cell indices in classification and treatment of anemias: from M.M. Wintrobes’s original 1934 classification to the third millennium. Curr Opin Hematol. 2013;20(3):222–30. doi: 10.1097/MOH.0b013e32835f5933 23449069

[35] Guyatt GH, Oxman AD, Ali M, Willan A, McIlroy W, Patterson C. Laboratory diagnosis of iron-deficiency anemia: an overview. J Gen Intern Med. 1992;7(2):145–53. doi: 10.1007/BF02598003 1487761

[36] Organization WH. Iron Deficiency Anaemia: Assessment, Prevention And Control. A guide for programme managers. WHO; 2001.

[37] Camaschella C. Iron-Deficiency Anemia. N Engl J Med. 2015;373(5):485–6. 10.1056/NEJMc1507104 26222573

[38] Cooper M, Greene-Finestone L, Lowell H, Levesque J, Robinson S. Iron sufficiency of Canadians. Health Rep. 2012;23(4):41–8. 23356044

[39] Jablonka A, Wetzke M, Sogkas G, Dopfer C, Schmidt RE, Behrens GMN, et al. Prevalence and Types of Anemia in a Large Refugee Cohort in Western Europe in 2015. J Immigr Minor Health. 2018. 10.1007/s10903-018-0725-6 29582203

[40] Ali MA, Luxton AW, Walker WH. Serum ferritin concentration and bone marrow iron stores: a prospective study. Can Med Assoc J. 1978;118(8):945–6. 647567 PMC1818245

[41] Thomas DW, Hinchliffe RF, Briggs C, Macdougall IC, Littlewood T, Cavill I, et al. Guideline for the laboratory diagnosis of functional iron deficiency. Br J Haematol. 2013;161(5):639–48. doi: 10.1111/bjh.12311 23573815

[42] Khuri J, Wang Y, Holden K, Fly AD, Mbogori T, Mueller S, et al. Dietary Intake and Nutritional Status among Refugees in Host Countries: A Systematic Review. Adv Nutr. 2022;13(5):1846–65. doi: 10.1093/advances/nmac051 35561746 PMC9526844

[43] UNHCR. World Food Programme: The UN Refugee Agency; 2022 [Available from: https://www.unhcr.org/world-food-programme-49eed2ba6.html].

[44] Alemayehu A, Gedefaw L, Yemane T, Asres Y. Prevalence, Severity, and Determinant Factors of Anemia among Pregnant Women in South Sudanese Refugees, Pugnido, Western Ethiopia. Anemia. 2016;2016:9817358. doi: 10.1155/2016/9817358 28058116 PMC5183745

[45] Hossain SM, Leidman E, Kingori J, Al Harun A, Bilukha OO. Nutritional situation among Syrian refugees hosted in Iraq, Jordan, and Lebanon: cross sectional surveys. Confl Health. 2016;10:26. doi: 10.1186/s13031-016-0093-6 27872656 PMC5111203

[46] Canada Go. #WelcomeAfghans: Key figures: Immigration, Refugees and Citizenship Canada; 2022 [Available from: https://www.canada.ca/en/immigration-refugees-citizenship/services/refugees/afghanistan/key-figures.html].

[47] Canada Go. Canada-Ukraine Authorization for Emergency Travel: Immigration, Refugees and Citizenship Canada; 2022 [Available from: https://www.canada.ca/en/immigration-refugees-citizenship/news/2022/03/canada-ukraine-authorization-for-emergency-travel.html].

[48] Abubakar I, Aldridge RW, Devakumar D, Orcutt M, Burns R, Barreto ML, et al. The UCL-Lancet Commission on Migration and Health: the health of a world on the move. Lancet. 2018;392(10164):2606–54. doi: 10.1016/S0140-6736(18)32114-7 30528486 PMC7612863

[49] Pottie K, Greenaway C, Feightner J, Welch V, Swinkels H, Rashid M, et al. Evidence-based clinical guidelines for immigrants and refugees. CMAJ. 2011;183(12):E824–925. doi: 10.1503/cmaj.090313 20530168 PMC3168666

[50] UNHCR. Figures at a glance 2019 [Available from: http://www.unhcr.org/statistics].

