# Supplementary figures and images for: Iron deficiency, anemia and association with refugee camp exposure among recently resettled refugees: A Canadian retrospective cohort study
Source: PLoS One. 2022 Dec 15;17(12):e0278838. doi: 10.1371/journal.pone.0278838 (PMC9754286; doi:10.1371/journal.pone.0278838)

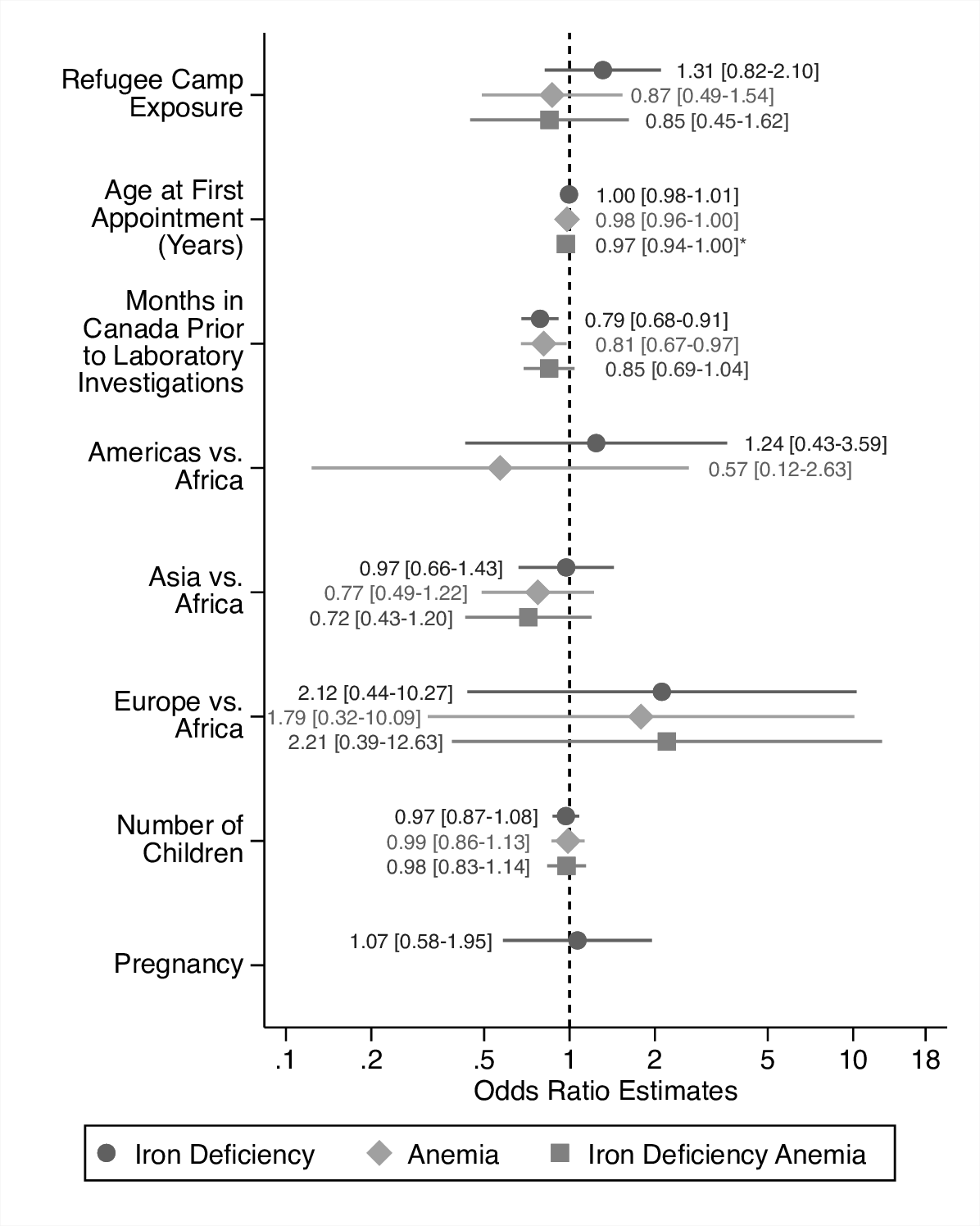

Supplement: S1 Fig — For sensitivity analysis, ID was defined as a serum transferrin saturation (tsat) ≤ 0.16 and IDA was defined as the presence of anemia as defined by WHO thresholds in combination with a tsat ≤ 0.16. Multi-variable logistic regression was used to estimate the odds of ID, anemia, and IDA in female refugees after adjusting for refugee camp exposure, age, global region of origin (defined by UN global regions), pregnancy status and number of children. (TIF) [file pone.0278838.s001.tif]
