# Supplementary material for: Iron deficiency, anemia and association with refugee camp exposure among recently resettled refugees: A Canadian retrospective cohort study
Source: PLoS One. 2022 Dec 15;17(12):e0278838. doi: 10.1371/journal.pone.0278838 (PMC9754286; doi:10.1371/journal.pone.0278838)
Supplement: S1 Table — (DOCX) [file pone.0278838.s002.docx]

| **Table S1. Sensitivity analysis; primary outcomes by sex and refugee camp exposure using serum transferrin saturation (tsat) (N = 1032)** | | | | | | |
| --- | --- | --- | --- | --- | --- | --- |
| **Variable** | **Female N = 534** | | | **Male N = 498** | | |
|  | **Refugee Camp +**  **N = 94** | **Refugee Camp -**  **N = 440** | **p-value** | **Refugee Camp +**  **N = 99** | **Refugee Camp -**  **N = 399** | **p-value** |
| **Iron Deficiency –**  **N (%)** | 40 (42.6) | 156 (35.5) | 0.20 | 2 (2.0) | 24 (6.0) | 0.13 |
| **Anemia – N (%)** | 19 (20.2) | 91 (20.7) | 0.99 | 4 (4.0) | 5 (1.3) | 0.08 |
| **Iron Deficiency Anemia – N (%)** | 14 (14.9) | 67 (15.2) | 0.99 | 0 (0) | 1 (0.3) | 0.99 |
