# Supplementary material for: Iron deficiency, anemia and association with refugee camp exposure among recently resettled refugees: A Canadian retrospective cohort study
Source: PLoS One. 2022 Dec 15;17(12):e0278838. doi: 10.1371/journal.pone.0278838 (PMC9754286; doi:10.1371/journal.pone.0278838)
Supplement: S2 Table — (DOCX) [file pone.0278838.s003.docx]

| **Table S2. Sensitivity analysis: Unadjusted outcomes among female refugees using transferring saturation (tsat) (N = 534).** | | | |
| --- | --- | --- | --- |
| **Variables** | **Iron Deficiency** | **Anemia** | **Iron Deficiency Anemia** |
|  | **Unadjusted Odds Ratio [95% CI]** | **Unadjusted Odds Ratio [95% CI]** | **Unadjusted Odds Ratio [95% CI]** |
| **Refugee Camp Exposure** | 1.35  [0.86 – 2.12] | 0.97  [0.56 – 1.69] | 0.97  [0.52 – 1.82] |
| **Age at First Appt.** | 0.99  [0.98 – 1.01] | **0.98**  **[0.96 – 1.00]*** | **0.96**  **[0.94 – 0.99]** |
| **Time (Months) in Canada Prior to Blood Work** | **0.80**  **[0.69 – 0.92]** | **0.83**  **[0.70 – 0.99]** | 0.87  [0.72 – 1.05] |
| **UN Global Region** | Calculated vs. Africa | Calculated vs. Africa | Calculated vs. Americas |
| Americas | 0.98  [0.35 – 2.74] | 0.47  [0.11 – 2.13] | NC** |
| Asia | 1.09  [0.76 – 1.57] | 0.86  [0.56 – 1.33] | 0.77  [0.47 – 1.25] |
| Europe | 1.35  [0.30 – 6.16] | 1.42  [0.27 – 7.53] | 1.94  [0.36 – 10.30] |
| **No. of Children** | 0.97  [0.88 – 1.06] | 0.93  [0.83 – 1.04] | 0.89  [0.78 – 1.02] |
| **Pregnancy** | 1.11  [0.62 – 1.98] | N/A | N/A |
