# Supplementary material for: Iron deficiency, anemia and association with refugee camp exposure among recently resettled refugees: A Canadian retrospective cohort study
Source: PLoS One. 2022 Dec 15;17(12):e0278838. doi: 10.1371/journal.pone.0278838 (PMC9754286; doi:10.1371/journal.pone.0278838)
Supplement: Attachment — Submitted filename: Response to reviewers - Sept 17.21.docx [file pone.0278838.s004.docx]

**Marta Davidson**, PhD MD FRCPC

Hematologist

Division of Medical Oncology and Hematology

Princess Margaret Cancer Center

700 University Ave, 6W091

Toronto, Ontario M5G 1Z5

Sept 17, 2021

Dear Editor:

Thank you for taking the time to review our manuscript entitled “*Iron deficiency, anemia and association with refugee camp exposure among recently resettled refugees: a Canadian retrospective cohort study*”, and for your insightful suggestions on how to improve it.

We have addressed your requirements as follows:

1. “*Please ensure that your manuscript meets PLOS ONE's style requirements, including those for file naming*”

- You will now find the manuscript is in compliance with *PLOS One* style requirements. A marked-up copy and a “clean” copy of the manuscript have been uploaded.

2. “*Please include captions for your Supporting Information files at the end of your manuscript, and update any in-text citations to match accordingly*”

- Captions for Supporting Information files have now been included at the end of the manuscript and in-text citations have been updated accordingly.

3. “*We note that you have indicated that data from this study are available upon request. PLOS only allows data to be available upon request if there are legal or ethical restrictions on sharing data publicly.”*

- We have obtained exceptional permission form our data custodian (The Mosaic Primary Care Network) outside of our existing research agreement to allow data-sharing of fully de-identified data with the journal to allow for review of our analysis and confirmation of the study results we present. The exceptional permission we received does not allow any posting or sharing of these data outside of the journal to with any other third parties outside of the journal however and we request that this is respected by the journal as any sharing would be a breach of the research agreement our study team has with our data custodian and would jeopardize our data access and multiple ongoing studies.
- To ensure our dataset was fully de-identified we had to remove 5 study participants who were originally presented in table cells with <5 participants and could allow for potential indirect identification and re-analyze the entire study cohort. We have updated our flow diagram (Figure 1), Table 1 and manuscript text to highlight this change. Our study findings did not change with this re-analysis.
- In addition to the final de-identified dataset, we have submitted with these revisions, we have also provided our raw data output from our analyses, and STATA code we developed to perform these analyses. We hope that providing our raw analyses will provide the reviewers confidence in our methodology and the results we present.
- For further inquiries regarding the data sharing restrictions please contact the data custodian’s privacy officer Dr. Valerie Fleisch ([privacy@mosaicpcn.ca](mailto:privacy@mosaicpcn.ca)).

4. “*Please review your reference list to ensure that it is complete and correct. If you have cited papers that have been retracted, please include the rationale for doing so in the manuscript text, or remove these references and replace them with relevant current references. Any changes to the reference list should be mentioned in the rebuttal letter that accompanies your revised manuscript. If you need to cite a retracted article, indicate the article’s retracted status in the References list and include a citation and full reference for the retraction notice.”*

- The reference list has been reviewed and is complete and correct. There are no references to retracted articles.

We hope that you will find our revisions satisfactory.

Sincerely,

Marta Davidson
